# Using large language models for enhanced fraud analysis and detection in blockchain based health insurance claims

**DOI:** 10.1038/s41598-025-15676-4

**Published:** 2025-08-13

**Authors:** Ruba Islayem, Senay Gebreab, Walaa AlKhader, Ahmad Musamih, Khaled Salah, Raja Jayaraman, Muhammad Khurram Khan

**Affiliations:** 1 Department of Computer & Information Engineering, Khalifa University, Abu Dhabi, UAE; 2 Department of Management Science & Engineering, Khalifa University, Abu Dhabi, UAE; 3 Department of Industrial Engineering, New Mexico State University, Las Cruces, NM USA; 4 Center of Excellence in Information Assurance, King Saud University, Riyadh, Saudi Arabia

**Keywords:** Fraud Detection, Health Insurance Claims, Blockchain, Large Language Model (LLM), Retrieval-Augmented Generation (RAG), Computer science, Information technology, Health care, Health services

## Abstract

Traditional health insurance claim processing systems are plagued by inefficiencies and vulnerabilities, often resulting in significant financial losses due to fraudulent activities. Existing fraud detection methods are largely manual, time-consuming, and inadequate for handling the complexity and scale of modern fraudulent schemes. Moreover, the trust-based relationships between insurers and healthcare providers lack mechanisms to ensure data integrity and prevent manipulation. While several blockchain-based systems have been proposed to improve transparency and tamper resistance, they typically focus on structured data and predefined fraud types, offering limited adaptability and analytical insight. This paper proposes a novel solution leveraging blockchain technology and Large Language Models (LLMs) to transform fraud detection. The system uses Ethereum smart contracts (SCs) to securely store medical records and claim details on a decentralized, tamper-proof ledger that ensures data integrity, traceability, and accountability. This immutable data is accessed by an LLM via a Retrieval-Augmented Generation (RAG) system, which enables intelligent retrieval and analysis of relevant clinical information to detect fraud patterns and inconsistencies. To support complex scenarios involving free-text documents, unstructured clinical data, such as lab reports, are stored using decentralized off-chain storage and retrieved during LLM analysis. In addition, an LLM-powered chatbot also allows insurance providers to interact with the system in natural language for claim inquiries, explanations, and summaries. The architecture, sequence diagrams, and implementation algorithms outline the development process, while testing scenarios demonstrate the system’s ability to detect fraud such as inflated costs, unnecessary treatments, and unrendered services. Evaluation using both synthetic and public clinical datasets showed strong performance, with the LLM achieving up to 99% fraud detection accuracy. Cost, security, and scalability analyses confirm the system’s practicality and resilience, with the complete detection process executing in just 13 seconds. By overcoming the limitations of traditional systems, this framework offers a scalable and adaptable approach for healthcare and other domains. The SCs and source code are publicly available on GitHub.

## Introduction

Health insurance claims are a fundamental component of the healthcare system, allowing patients to access necessary medical services while insurance providers reimburse healthcare costs according to agreed-upon policies^1^. These claims cover a wide range of medical expenses, including consultations, treatments, medications, and surgeries. Insurance companies process these claims to ensure that payments align with policy terms before reimbursing healthcare providers. However, the healthcare insurance industry faces the escalating challenge of fraudulent activities that inflate costs and impose significant burdens on both insurers and honest policyholders. Health insurance fraud involves the deliberate provision of false or misleading information to obtain unauthorized benefits or payments^2^. This can take many forms, such as billing for services not rendered, inflating costs, duplicating claims, and prescribing unnecessary medications or procedures^3^. The consequences are far-reaching, driving up premiums, causing financial losses for insurers, and restricting access to care for legitimate patients. Globally, healthcare fraud costs billions annually. In 2019, the U.S. Department of Health and Human Services reported losses of $2.6 billion from fraudulent claims^4^. By 2020, this figure had risen to $6 billion across federal healthcare programs and private insurers, with fraud cases involving 345 individuals in 51 federal districts^5^. These figures underscore the pervasive nature of health insurance fraud and its severe impact on healthcare systems.

**Fig. 1:**
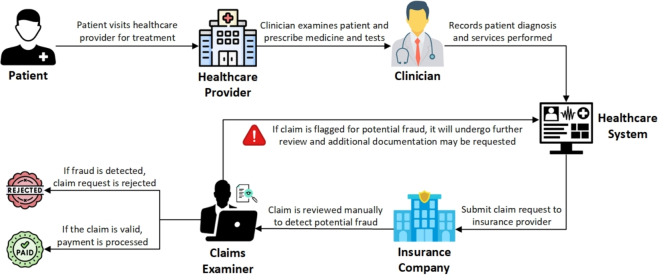
Flow diagram illustrating the typical process for health insurance claim submission and review.

When a patient visits a healthcare provider, a health insurance reimbursement claim is initiated, as shown in Fig. 1. The clinician evaluates the patient’s condition, recommends treatments and tests as needed, and documents the diagnosis and services provided in detail before compiling all this information into a claim request to be sent to the insurance company^6^. Afterward, the healthcare provider submits the claim request to seek reimbursement from the insurance company for the provided services. Upon receiving a claim request, the insurance company evaluates adherence to policy regulations by meticulously verifying the submitted information against the policy terms^7^. If discrepancies or suspicious elements arise, such as inconsistencies between billed services and accompanying documentation, the claim is flagged for review, and additional documentation is requested from the healthcare provider. Once all required information is collected and compiled, the claim is either approved if legitimate or rejected if signs of fraud are detected.

This complex and demanding process highlights the challenges insurance companies face in handling claims and detecting fraud, as claims are manually examined by human representatives following policy rules. This laborious method can be error-prone and time-consuming when dealing with several claims. Additionally, the conventional system lacks authentication mechanisms, as it heavily relies on trust between healthcare providers and insurers. The lack of solid measures creates challenges in upholding data accuracy, accountability, and transparency, leading to a higher risk of claim tampering or falsification. Moreover, insurers often lack access to essential records, such as medical reports and prescriptions, making it difficult to verify the legitimacy of claims. Especially with discrepancies that require additional documentation from healthcare providers, which leads to further delays and inefficiencies^3^.

Therefore, integrating advanced technologies such as blockchain and Large Language Models (LLMs) offers significant advantages for enhancing health insurance claims processing and fraud detection^8,9^. Blockchain provides a tamper-proof ledger that ensures traceability, integrity, and accountability throughout the claims process^10^. By recording every transaction in a decentralized manner, it eliminates the possibility of data manipulation, allowing insurers to trust the information they receive. Blockchain also enables tracking of all transactions and modifications, providing a comprehensive log of who added records and made actions^11^. Moreover, LLMs are advanced models specifically designed to process and generate human-like text based on the context provided^12^. They excel at analyzing vast amounts of data, which enables them to quickly identify patterns and anomalies indicative of fraudulent activities. Their natural language processing capabilities allow for the interpretation of unstructured textual data, such as clinical notes and test reports, thereby enhancing the accuracy of fraud detection. Additionally, Retrieval-Augmented Generation (RAG) significantly enhances the performance of LLMs by providing access to external knowledge sources, enabling the generation of personalized and contextually relevant responses based on the provided knowledge base. Therefore, these technologies together streamline the claims management process and minimize the risk of fraud.

In this paper, we propose a novel solution that combines blockchain technology and LLMs to improve efficiency and accelerate fraud detection in health insurance claims. Our system enables a tamper-proof and reliable documentation system that employs a blockchain-based framework to securely track and record transactions related to medical records and claim requests to guarantee traceability, integrity, and accountability. While previous blockchain-based systems have enhanced transparency and ensured data integrity, they are often limited to structured data and lack mechanisms for real-time analysis of unstructured medical records or support for interactive, context-aware decision-making. Unlike traditional rule-based or machine learning approaches, our system introduces a RAG reasoning mechanism where blockchain transaction data is dynamically retrieved, processed, and analyzed using an LLM. This integration enables real-time detection and flagging of various types of fraudulent claims based on contextual understanding and natural language reasoning. An LLM-powered chatbot further enhances the system by facilitating user interactions and providing context-aware explanations and responses for claim inquiries. Our system offers robust features by leveraging blockchain and LLMs, including analytical insights, reasoning, detailed explanations, summaries, and improved fraud detection capabilities.

The main contributions of this paper are summarized as follows:

- We propose a blockchain and LLM architecture that enables intelligent, context-aware fraud analysis and detection in real-time, addressing key challenges in trust, auditability, and automation in insurance workflows.
- We develop a tamper-proof framework using Ethereum smart contracts (SCs) to securely store claim-related data on a decentralized, immutable ledger. The framework ensures data integrity, enforces role-based access control, and enables transparent auditing. It also supports both structured and unstructured clinical data through decentralized off-chain storage (IPFS), allowing this data to be retrieved and analyzed by the LLM for fraud detection.
- We introduce a novel fraud detection mechanism where an LLM-based agent, enhanced with RAG capabilities, retrieves and analyzes trusted blockchain transactions in real-time to detect inconsistencies and patterns of fraudulent activity.
- We develop a chatbot interface that allows insurance providers to interact with the LLM in natural language to inquire about claim details, investigate potential fraud, and receive clear explanations and summaries.
- We evaluate the effectiveness of the proposed system using both synthetic and public clinical datasets, achieving up to 99% fraud detection accuracy with perfect precision (100%) and strong fraud-type classification performance.
- We present a detailed explanation of the system architecture and algorithms developed to implement our solution. In addition, we evaluate the system performance through testing scenarios and conduct cost, security, and scalability analyses, achieving an end-to-end detection time of 13 seconds.

## Related work

This section provides an overview of existing literature and solutions for health insurance fraud detection systems, specifically focusing on solutions that integrate blockchain, artificial intelligence (AI), and machine learning (ML) technologies to enhance the efficiency and accuracy of fraud detection processes.

### Blockchain-based solutions for health insurance claims management and fraud detection

Several studies have proposed blockchain-based systems that aim to enhance health insurance processes and improve fraud detection. By leveraging blockchain and SCs, researchers have developed solutions that facilitate secure data sharing and ensure the integrity, trustworthiness, and transparency of medical records and claim details.

Early efforts, such as the work by the authors in^13^, introduced secure frameworks for managing the health insurance claims process using blockchain and the InterPlanetary File System (IPFS) for decentralized storage of large files. This system enables healthcare providers and insurers to exchange verified claim information in a trusted environment, where SCs record each transaction immutably. Similarly, Liu *et al.*^14^ proposed a cross-institutional blockchain system that connects insurance agencies, hospitals, and government departments. By interlinking three distinct blockchains, their solution allows for comprehensive verification of medical records and treatment histories during the claims process. This approach facilitates fraud detection through multi-source validation. Expanding on this multi-chain concept, Mahapatra *et al.*^15^ presented a system that separates responsibilities across three blockchains: e-chain for encrypted health records, d-chain for diagnoses, and i-chain for insurance claim handling. Patients upload encrypted EHRs to IPFS and store hash references on-chain, while insurers validate claims by cross-checking related entries across all chains. This approach enhances fraud detection by identifying inconsistencies among patient-reported treatments, actual medical services, and claim submissions. Additionally, a layered architecture was proposed in^16^, where SCs embedded in the blockchain layer autonomously manage healthcare records, verify authorizations, and process claim transactions. This architecture ensures end-to-end traceability and facilitates automatic fraud detection through rule-based contract logic. Alnuaimi *et al.*^17^ proposed a private Ethereum-based prescription claims system that incorporates SCs for managing approvals and IPFS for secure document handling, combined with a gateway-based access control mechanism to preserve privacy. Their design enhances traceability and data immutability while preventing unauthorized claims or retroactive data manipulation. Similarly, Kaafarani *et al.*^18^ developed a SC-driven taxonomy of insurance fraud scenarios and used a platform recommender system to evaluate optimal blockchain deployment environments. Their experiments with Hyperledger Fabric demonstrated the system’s ability to efficiently execute contract-based fraud checks without external analytics. Furthermore, Krishna *et al.*^19^ introduced MedBlockSure, a permissioned blockchain system where SCs automate claim verification and eliminate intermediaries. This architecture synchronizes patient, hospital, and insurer records to reduce opportunities for duplicate or falsified claims. At a national scale, Alamsyah and Setiawan^20^ implemented a blockchain model for public health insurance claims. They employed SCs to enforce access control and immutably log every claim-related action. Their system improves auditability and accountability while protecting patient data from tampering or unauthorized viewing.

These blockchain-based approaches demonstrate the capability of SC-based systems to autonomously verify, store, and track insurance claims. By eliminating manual gatekeeping, enforcing transparency, and preserving data provenance, such systems establish a robust foundation for detecting and preventing healthcare insurance fraud at scale.

### AI and ML enabled blockchain-based solutions for insurance fraud detection

Various studies have highlighted the potential of combining AI and ML predictive analytics with the transparency and security of blockchain to build robust and trustworthy health insurance fraud detection systems. Studies have explored the use of AI and ML to detect fraudulent activity in health insurance claims. These approaches often leverage structured claim data to identify anomalous patterns and behaviors indicative of fraud. Hamid *et al.*^21^ proposed a hybrid method combining association rule mining with anomaly detection techniques such as Isolation Forest and One-Class SVM to uncover irregular claim patterns. Similarly, Nabrawi and Alanazi^2^ applied classical supervised learning methods, including logistic regression and random forest, to classify claims as fraudulent or legitimate using real-world insurance data. Baruah *et al.*^22^ further demonstrated that integrating expert-defined fraud triggers into models like decision trees and gradient boosting can significantly enhance detection performance by combining domain knowledge with machine learning. Parthasarathy *et al.*^23^ employed a Bayesian-optimized XGBoost model to process large-scale insurance claim datasets. Their approach showed that well-tuned ensemble methods can effectively identify suspicious behavior while maintaining model interpretability.

Building on the strengths of traditional ML techniques, other studies have explored their integration with blockchain technology to enhance data integrity, traceability, and trust. The authors in^24^ reviewed the potential of AI and blockchain to enhance fraud detection in insurance claims. They examined how AI can analyze data to detect suspicious patterns, while blockchain’s secure and immutable framework can improve data integrity and trust in transactions. Vyas *et al.*^25^ and Alnavar *et al.*^26^ presented an architecture combining blockchain technology with ML to detect fraudulent insurance claims. These systems leveraged blockchain to create a secure, transparent platform for data sharing among insurance participants. The shared data is then used to train ML models for enhanced fraud detection accuracy. In both studies, patient records and claim data are stored within SCs, and multiple ML algorithms are evaluated, with Random Forest achieving the highest accuracy rates. Agarwal in^27^ proposed an intelligent ML approach for detecting fraudulent medical insurance claims using the K-means clustering algorithm. This unsupervised learning technique groups similar claims together based on shared features, such as claim amount and procedure code. By analyzing these clusters, the algorithm can identify outliers that deviate significantly from the norm, which indicates fraudulent activity. Additionally,^28^ introduced a hybrid system where decision tree-derived fraud rules are embedded directly into SCs. The approach begins by using decision tree algorithms to classify a dataset of original health insurance claims and extract patterns of fraudulent activities. The extracted classification rules are then programmed into a SC to automate the detection and prevention of fraud during claims processing.

### Research gaps

Recent literature has shown progress in applying blockchain and ML technologies to health insurance fraud detection. Several blockchain-based systems have been proposed to ensure secure, transparent claim processing using SCs, decentralized storage, and tamper-proof ledgers. In parallel, traditional ML methods, such as decision trees, random forests, clustering, and rule-based classification, have been applied to detect fraudulent patterns using structured claim data. Moreover, some recent works have attempted to combine blockchain with ML to benefit from both secure data integrity and predictive analytics. However, despite these advancements, several critical research gaps remain unaddressed.

First, the majority of existing systems rely exclusively on structured input data, which significantly limits their ability to detect fraud in real-world scenarios where claim records often include unstructured formats such as free-text notes, scanned documents, or physician narratives. These systems lack the capability to interpret and analyze natural language content, thereby overlooking fraud that may be embedded in textual evidence.

Second, most current solutions, whether blockchain-based, ML-driven, or hybrid, do not support real-time or automated analysis. Fraud detection processes are often delayed or dependent on manual review, resulting in slower detection and response. Particularly, there is a lack of frameworks that dynamically retrieve and process data from on-chain transactions and external records in real time.

Third, while traditional ML models have proven effective in detecting common fraud types, such as inflated billing and duplicate claims, they are generally rule-based and limited in their ability to generalize across diverse or complex fraud scenarios. This is especially true for detecting complex fraud patterns such as unrendered services or medically unnecessary procedures, which require contextual understanding of clinical records.

Lastly, there is a clear absence of systems that incorporate intelligent user interaction. Current platforms typically lack interfaces that allow insurance analysts to query, explain, or interpret claim decisions using natural language. The absence of such interaction restricts the transparency and usability of fraud detection systems in real-world insurance workflows.

These gaps highlight the need for a more intelligent, adaptable, and explainable fraud detection framework. Our proposed system addresses these limitations by integrating blockchain with a RAG framework. This architecture enables secure on-chain storage of patient and claim data, real-time fraud analysis of structured and unstructured data, and intelligent user interaction through a natural language chatbot. It provides a unified platform for interpretable and context-aware fraud detection, covering a broader range of fraud types with improved efficiency and accessibility.

## System design

In this section, we present our proposed system architecture, which details the relationships and roles of its key components. In addition, we include sequence diagrams to illustrate the process flow and interactions among these components.

### System architecture and components

**Fig. 2:**
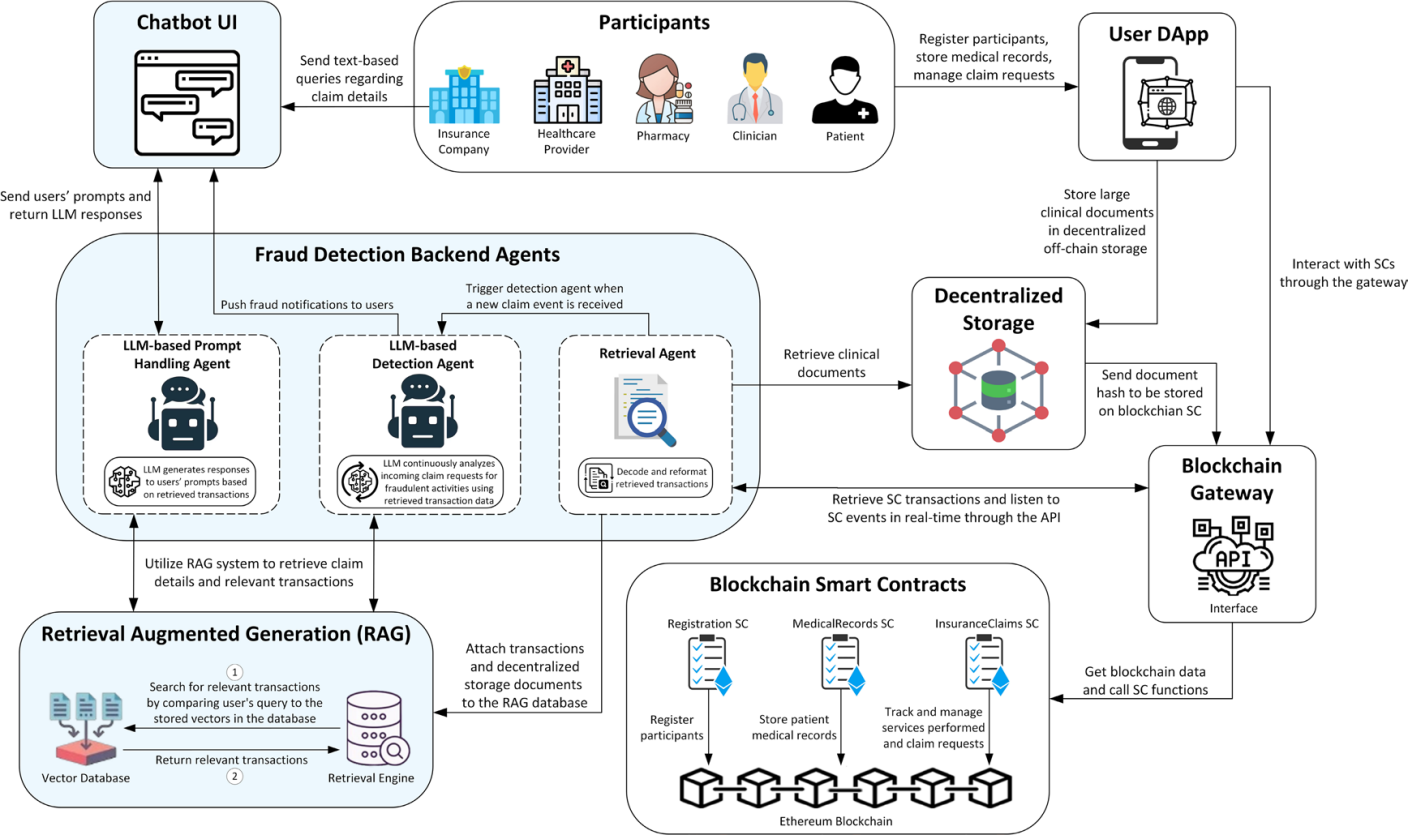
System architecture illustrating key components and their interactions in the proposed LLM and blockchain-based health insurance fraud detection system.

The system architecture presented in Fig. 2 illustrates the key components and their interactions within our proposed solution. It leverages blockchain SCs and decentralized storage for secure data storage and LLM-based agents with a RAG system for fraud detection and user interactions. Moreover, a blockchain gateway enables real-time access to blockchain data, while a chatbot interface facilitates user interactions through natural language. The following descriptions outline the role of each component in the system architecture.

**Participants:** The actors participating in our system include patients, clinicians, pharmacies, healthcare providers, and insurance companies. Each participant must register in the system to gain access to SC functions. All actors interact with the decentralized application (DApp) to call SC functions and upload documents to decentralized storage, while insurance providers can use the chatbot interface to access LLM-based features.

**Blockchain smart contracts:** The blockchain serves as the underlying layer for data storage in our system, which ensures data integrity, immutability, and transparency. SCs are programmable agreements that automatically execute predefined actions when specific conditions are met^29^. They enable secure interactions and automate various processes within the system. We have developed three blockchain SCs to manage and store patient medical records and claim requests. The Registration SC handles user registration and assigns roles to users to control access to specific SC functions. The MedicalRecords SC stores patient records and medical history, including details of doctor visits, diagnoses, tests, and decentralized storage hashes. The InsuranceClaims SC manages the claim process by recording all rendered services and dispensed prescriptions, submitting claim requests, and approving/rejecting claims. Together, these SCs ensure full traceability and provide the LLM with reliable data to enable efficient fraud detection and identification of responsible actors.

**Decentralized storage:** A decentralized off-chain storage, such as the InterPlanetary File System (IPFS), is used to handle large or unstructured clinical documents that cannot be stored directly on-chain due to size and cost constraints. When a user uploads a document, such as a test report or clinical note, it is stored in a decentralized off-chain system, and a content-addressed hash is generated. This hash is then recorded on the blockchain via SCs to ensure data integrity and traceability. During fraud analysis, the system can retrieve the document using the stored hash, allowing the LLM to reason over both structured on-chain data and unstructured clinical content.

**Blockchain gateway:** A blockchain gateway explorer, such as Etherscan, is a specialized tool that allows users to interact with the SCs and retrieve indexed blockchain data. We utilize the application programming interface (API) provided by the blockchain gateway to retrieve indexed transactions from the SCs. Moreover, we use the API to listen to SC events to enable real-time fraud detection capabilities.

**User DApp:** The user DApp enables users to call and execute SC functions through a user-friendly interface. It is built on top of the Ethereum network and interacts with deployed contracts using the blockchain gateway, which provides real-time access to SC functions and on-chain data. The DApp allows users to perform authorized actions such as registering patients, submitting medical records, adding services/prescriptions, and initiating or reviewing claim requests. All transactions are executed transparently on the blockchain and signed by the user’s wallet to ensure authorization and traceability. Moreover, the DApp also enables users to upload unstructured or large clinical documents to decentralized off-chain storage.

**Chatbot UI:** The chatbot serves as the main user interface (UI) that facilitates interaction between insurance providers and the LLM-based system. Through this interface, insurance providers can send natural language queries to obtain claim details, access patient records, and investigate potentially fraudulent claims. Additionally, they receive notifications through this interface whenever the LLM-based detection agent identifies new instances of fraud.

**Fraud detection backend agents:** The backend system consists of three agents: the retrieval agent, the detection agent, and the prompt handling agent. The retrieval agent is responsible for monitoring SC events in real-time to retrieve blockchain transactions and feed the retrieved data to the RAG component for ingestion. In addition to on-chain data, the retrieval agent also accesses decentralized off-chain storage to retrieve unstructured clinical documents using hashes stored in SCs. When a new claim request event is received, the retrieval agent triggers the detection agent to analyze the claim. The detection agent uses the RAG system to fetch relevant data for the claim evaluation, which allows the LLM to assess it for potential fraud. If the fraud detection agent suspects any fraudulent activity, it sends a notification to insurance providers through the chatbot UI. The prompt handling agent facilitates intelligent interactions by processing user prompts. It utilizes the RAG system to retrieve relevant transactions based on user input and leverages the LLM to generate responses using the retrieved data as context. This LLM-powered agent enhances the system’s capabilities by providing advanced features, including natural language understanding, data analysis, explanation generation, and claim summarization.

**Fig. 3:**
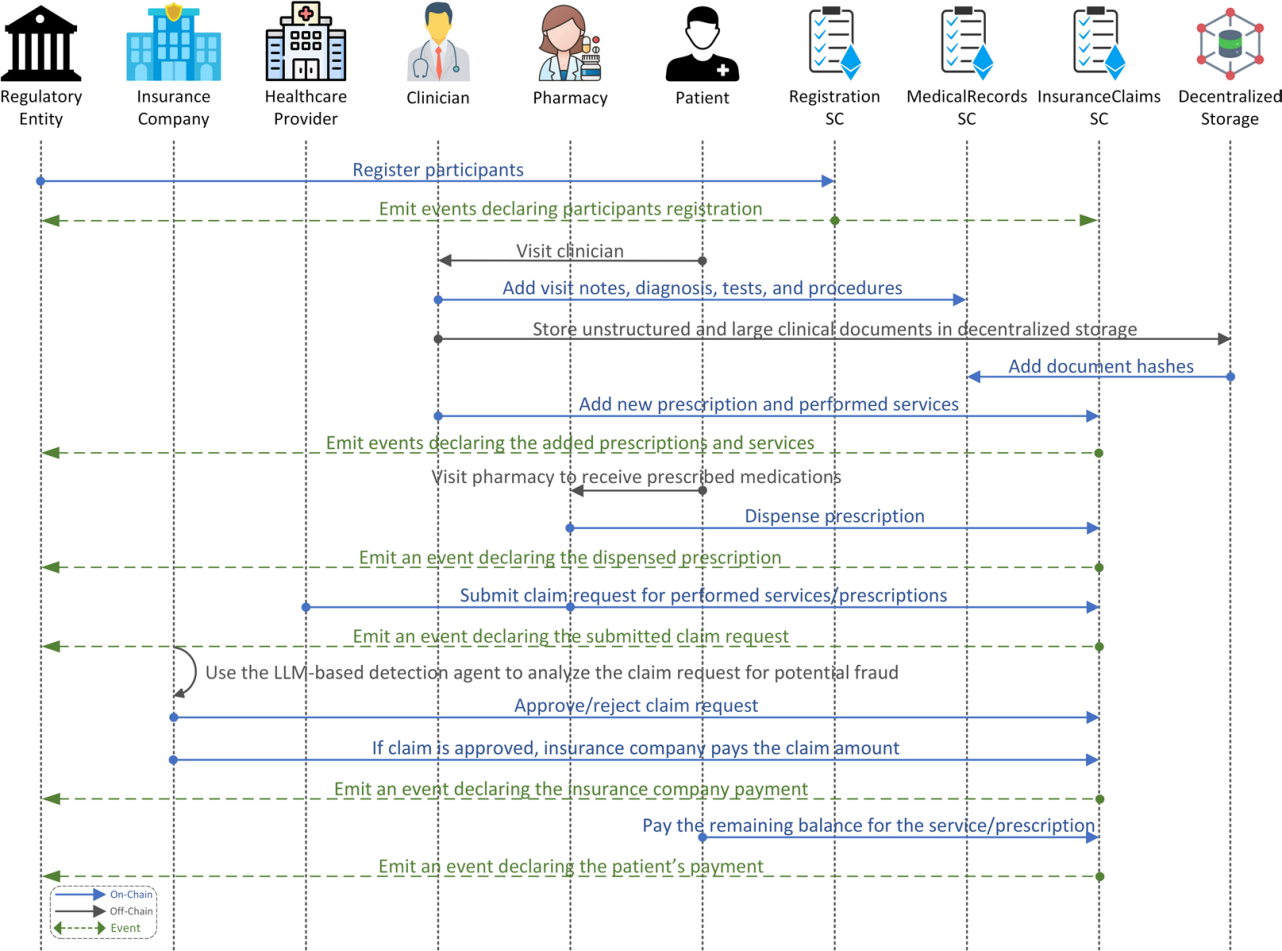
Sequence diagram depicting participants’ interactions with SCs for storing medical records and managing claim requests.

**RAG:** The RAG component plays a critical role in enhancing the capabilities of the LLM by providing contextual access to external data, such as blockchain transaction data. It stores transactions and clinical documents as embeddings in a vector database, allowing the prompt handling agent and the fraud detection agent to retrieve relevant data whenever needed. When the retrieval agent attaches transactions to the RAG component, each source document is split into smaller chunks. Each chunk is then converted into embeddings using a pre-trained embedding model and stored in a vector database. To retrieve relevant information, such as medical records and claim details, the retrieval engine performs a similarity search within the vector store to find relevant transaction data based on the received query and returns them to the agents for further processing by the LLM. For user queries, this enables the prompt handling agent to generate responses grounded in specific transaction details and medical records. For fraud detection, it allows the detection agent to analyze new claims within the context of historical transaction patterns and related patient data.

### Interactions and process flow

This subsection presents sequence diagrams that illustrate the relationships and interactions among the system components. Figure 3 illustrates the process of interacting with SCs to store medical records and manage claim requests. All actors must be registered on the system to participate. The regulatory entity initiates the registration process by calling functions from the Registration SC to register all participants, with events emitted to confirm each registration. When a patient visits a clinician for treatment, all relevant medical records, including visit notes, diagnoses, tests, and procedures, are stored on the blockchain through the MedicalRecords SC functions. For unstructured and large clinical documents, the files are stored in decentralized off-chain storage, and their corresponding content hashes are recorded on-chain via the MedicalRecords SC. Prescribed medications and performed services are also recorded via the InsuranceClaims SC, with events emitted to update the patient’s record with the added services and prescriptions. Following this, the patient visits a pharmacy to receive prescribed medications, after which the healthcare provider and pharmacy submit new claim requests to the insurance company. An event is emitted for each claim request, and the fraud detection agent uses the integrated LLM service to analyze these requests. The insurance company reviewers can then approve or reject the claim requests based on this analysis. If the claim is approved, the insurance company pays the claim amount, while patients cover any remaining balance for services or prescriptions not fully covered by the insurance provider.

**Fig. 4:**
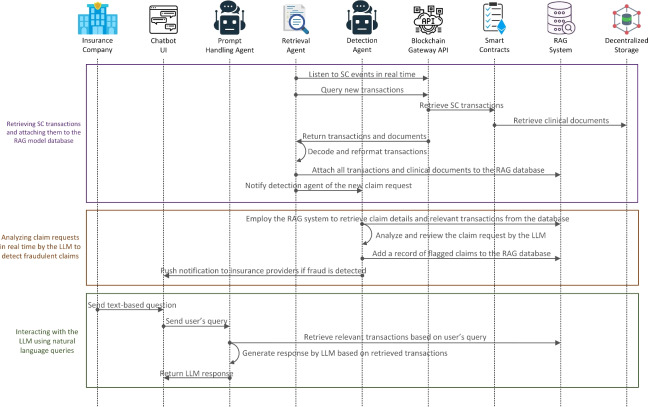
Sequence diagram illustrating SC transactions retrieval, real-time fraud detection, and LLM-user interactions.

Figure 4 illustrates the process of retrieving transactions from SCs, detecting fraudulent claims in real-time, and interacting with the LLM agents. The retrieval agent listens for SC events in real-time through the blockchain gateway. When a new claim request event is emitted, it queries the blockchain gateway to fetch the relevant transactions. These transactions are decoded, reformatted to extract the necessary data, and organized into JSON files. In cases where IPFS hashes are present, the retrieval agent also fetches the associated off-chain clinical documents from decentralized storage. Both the on-chain transaction data and the retrieved documents are then chunked into appropriate segments, converted into embeddings, and stored in the vector database within the RAG system. Upon receiving the new claim request, the retrieval agent invokes the detection agent, which uses the RAG system to retrieve relevant claim details and context, allowing the integrated LLM to analyze the claim for potential fraud. If fraud is detected, the detection agent flags the claim and passes it to the RAG system for storage. It then sends a notification to the insurance provider through the chatbot UI. Subsequently, the flagged claim can be reviewed by the insurance company reviewer, who can interact with the chatbot to obtain further insights and details. When the reviewer sends a text query to the chatbot, it is forwarded to the prompt handling agent, which utilizes the RAG system to retrieve relevant transactions from the vector database. The LLM then processes the query based on the retrieved data to generate a response and send it back to the user.

## Implementation details

This section details the implementation of our proposed system, including algorithms and descriptions of the developed SCs, the transaction retrieval service, and the integrated LLM and RAG models for fraud detection and user interactions. The SCs and the system source code are publicly available on GitHub at: https://github.com/rubak3/InsuranceFraudDetection.

### Blockchain and SCs implementation

To ensure transparency, immutability, and rule-based enforcement of sensitive operations such as claim processing, we implemented three Ethereum SCs as the core logic layer of the system. Ethereum SCs are chosen for their ability to automatically validate and enforce fraud prevention rules in a tamper-proof and decentralized manner. The developed SCs facilitate user registration, store medical records and services, and manage claim requests. The contracts are written in Solidity, which is a programming language specifically designed for Ethereum SCs, and they are developed using the Remix IDE. Remix is a powerful tool that provides an intuitive interface for coding, testing, and debugging SCs^30^. Here, we present detailed algorithms that demonstrate the functionality and underlying logic of our SCs.

Participants are initially registered through the Registration SC, where the contract owner assigns their roles to grant access to relevant SC functions. Patients are registered with details such as their Ethereum address, medical record number (MRN), and insurance provider address. All other participants follow a similar registration process, with mappings used to store and manage data for each participant type. This role-based registration approach ensures that only verified entities can interact with specific SC functions, thereby reducing the risk of unauthorized actions and supporting accountability. Once all participants are registered, MedicalRecords SC is used to store patient medical records.

Algorithm 1 outlines the process for adding patient medical records, including doctor visits, test results, diagnoses, and IPFS hashes. When a patient visits a clinician, the clinician calls the *addDoctorVisit* function to record the visit details in the *patientRecords* mapping. To add a test result, the *addLabResult()* function is called to add the patient’s address, the test Current Procedural Terminology (CPT) code, and the test result IPFS hash to the *patientRecords* mapping. CPT codes are numeric codes used to describe medical, surgical, and diagnostic services to ensure unified documentation^31^. Similarly, chronic disease diagnoses and surgical procedures are logged through *addChronicDisease()* and *addSurgery()* functions, which add the disease surgery codes to the patient records.

Algorithm 2 outlines the process of adding performed services and prescriptions via the InsuranceClaims SC. Rendered services such as doctor visits, lab tests, and procedures are recorded using the *addService()* function. This function is restricted to the clinician who performed the service. It updates the *services* mapping with the patient and clinician addresses, the service CPT code and cost, and a boolean value indicating whether a claim has been requested for the service. These details are essential to ensure that conditions are met when submitting claim requests. Similarly, the *addPrescription()* function is called to add new prescriptions by providing the patient’s address and the medication National Drug Code (NDC). Finally, when pharmacists dispense prescribed medications to patients, the *dispensePrescription()* function is called to record this action. The pharmacist provides the prescription ID and the cost, and the function verifies that the prescription has not been dispensed yet to update the *prescriptions* mapping. These functions are essential for a secure and trustworthy recording of all performed services and dispensed prescriptions to ensure that claim details are valid and accurate and prevent fraudulent claims.

**Algorithm 1:**
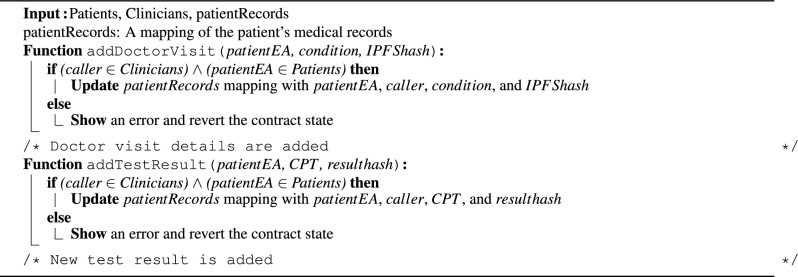
Storing patient medical records.

**Algorithm 2:**
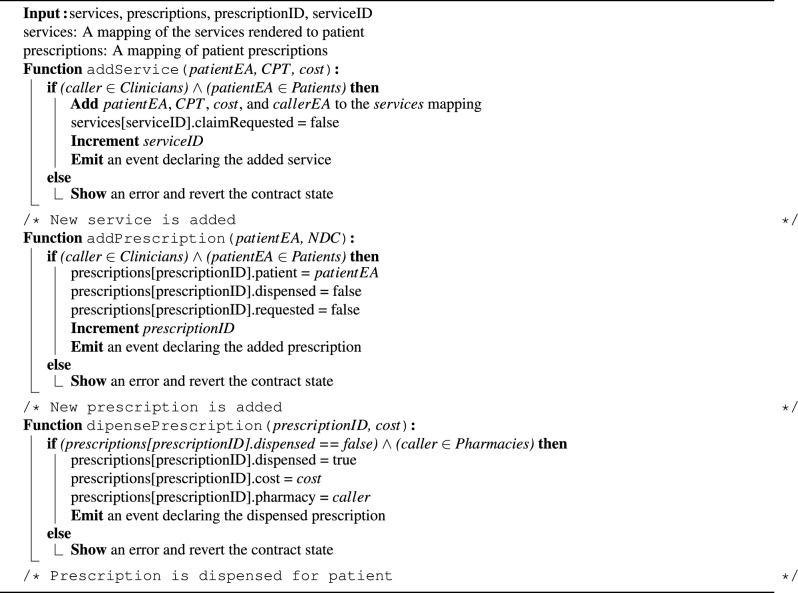
Adding services and prescriptions.

Algorithm 3 outlines the process of submitting new claim requests by hospitals and pharmacies. The *submitClaimRequest()* function is used to submit a claim for a performed service or dispensed medication. This function takes the patient’s address, service/prescription ID, and claim amount as inputs. First, it verifies that the claim request is submitted by the hospital where the service was performed. It then checks that no prior claim request exists for the same service/prescription, and ensures the claim amount does not exceed the total service/prescription cost. If these conditions are met, the *claimRequests* mapping is updated with all relevant details, and the claim status is initially set to “Pending”. The *services* mapping is also updated to set “claimRequested” to “true”, to prevent duplicate claims for the same service. Lastly, if the claim is approved following the LLM and reviewers’ analysis, the insurance company initiates the payment process by calling the *payClaim()* function. This function verifies that the caller and receiver addresses match the details associated with the specified claim ID. Additionally, the claim status must be “Approved,” and the payment amount must match the claim amount. If these conditions are met, the Ether amount is transferred to the receiver, the claim request status is changed to “Paid”, and an event is emitted.

**Algorithm 3:**
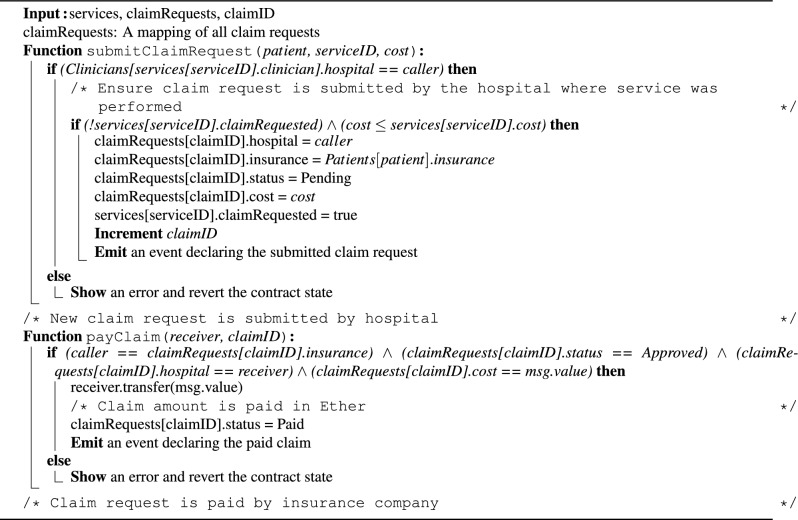
Submitting and paying claim requests.

### Transactions retrieval agent implementation

The fraud detection backend agents and chatbot are developed using JavaScript. The Etherscan API is utilized to retrieve transactions from the SCs, which is a widely used service that provides access to Ethereum blockchain data^32^. Etherscan is specifically chosen for its capability to query indexed transaction details, such as timestamps, function calls, and event logs, without running a full Ethereum node. This offers a lightweight and effective mechanism for real-time monitoring of blockchain activity, which is essential for prompt fraud detection. The process of retrieving SC transactions is outlined in Algorithm 4. A GET request is sent to the Etherscan API URL, including the SC address and the starting block number. The response returns all transactions associated with the specified SC. Each transaction is then decoded and formatted using the *formatTransactions()* function, which relies on the contract’s Application Binary Interface (ABI) to convert the input data back to its original textual format. Key fields such as the timestamp, function name, input parameters, and event logs are extracted from each transaction and attached to the RAG system as a knowledge source. This textual data provides essential context for the LLM to understand and analyze claim details. Lastly, the block number of the most recently retrieved transaction is stored to ensure that future calls retrieve only new entries. Moreover, when transactions include IPFS hashes referencing unstructured clinical documents, the system issues a separate request to retrieve the corresponding file from the IPFS using a public IPFS gateway. The retrieved document is then parsed and included in the RAG database alongside on-chain data. This integration allows the LLM to reason over both blockchain-stored metadata and associated off-chain clinical content to significantly improve fraud detection coverage. This retrieval function is triggered whenever a new claim request is received, allowing updated and real-time transactions to be available to the detection agent through the RAG system.

**Algorithm 4:**
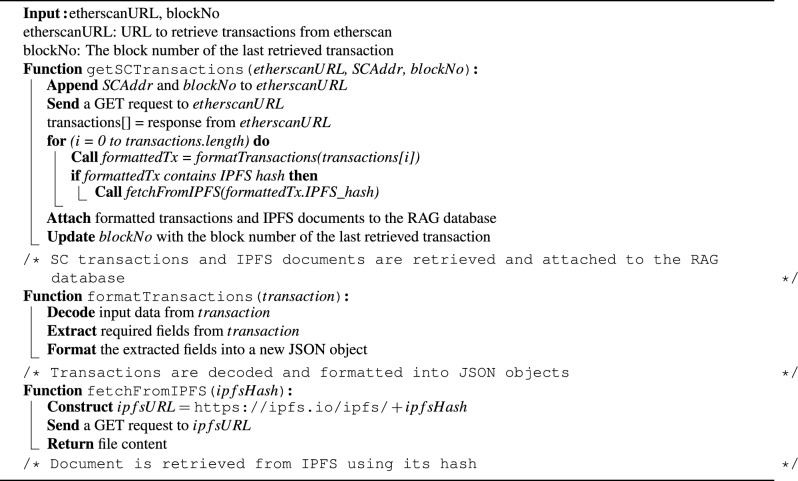
Retrieving SC transactions and IPFS documents.

### Implementation of LLM and RAG for user interactions and fraud detection

To enable intelligent user interactions and real-time fraud detection, we employ an OpenAI assistant configured with GPT-4o as the LLM base model. GPT-4o is selected for its strong performance in tasks involving reasoning, context understanding, and decision support, making it well-suited for analyzing complex clinical and transactional data^33–35^. In our implementation, OpenAI’s built-in file search tool is used to provide the RAG functionality^36^. RAG capability is essential to ensure that LLM responses are grounded in verifiable blockchain transactions, rather than relying solely on the model’s internal knowledge, thus enhancing accuracy and relevance. The assistant is also configured with a function calling tool, which enables automated execution of SC functions based on user requests. These tools improve the system’s usability and interactivity by allowing users to query claim details, receive contextual explanations, and even approve or reject claims through natural language requests.

The system interfaces with the LLM model through the OpenAI API, handling both user conversations and SC function execution. Initially, a new assistant is initialized with system instructions and a linked vector store, which stores transactions retrieved through the *getSCTransactions* function in Algorithm 4. In this case, the OpenAI assistant handles the indexing and similarity search internally. Algorithm 5 details the process of interacting with the AI assistant. User messages are sent to the assistant with the “user” role. The assistant utilizes the file search tool and performs a similarity search on the indexed transaction files to retrieve contextually relevant information for the user query. The retrieved context is then used by the LLM to generate a relevant response. The system reads the assistant’s output and displays the response in the chatbot interface once a message with the “assistant” role is received. On the other hand, when the user initiates an action, such as approving a claim, the assistant detects the request, identifies the intended function and its parameters, and passes them to the backend. Using the Ethers JavaScript library, the system creates an instance of the SC and calls the specified function. The user is prompted to sign the transaction through the connected wallet, and the resulting transaction hash is returned. The user signature is essential here as part of access control measures to verify that the user is authorized to call this function.

**Algorithm 5:**
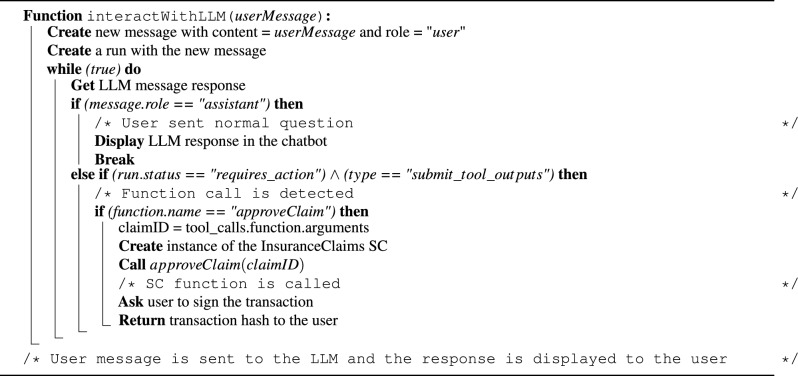
Interacting with the LLM.

Algorithm 6 outlines the process for real-time fraud detection in submitted claim requests. The system continuously listens for new events emitted by the SC using the *On()* function provided by the Ethers library, which returns the specified event details whenever it is emitted. This event-driven architecture enables immediate response to new claim submissions and ensures that potential fraud is identified at the earliest possible stage. When a *claimRequestSubmitted* event is emitted, the system retrieves recent transactions using the *getSCTransactions()* function from Algorithm 4. These transactions are decoded and uploaded to the OpenAI assistant, which handles indexing for subsequent retrieval. Next, the *interactWithLLM()* function from Algorithm 5 is invoked to send a message to the assistant, prompting it to review and analyze the claim for potential fraud. A binary output is used (1 for fraud detected, 0 for no fraud) to simplify decision integration and reduce latency in high-volume settings. An LLM is used in this step due to its ability to interpret textual clinical and transactional data, offering more flexibility and interpretability than fixed rule-based systems. If the response is 1, the claim is flagged as suspicious and stored in the assistant’s database. A notification is also sent to the insurance company through the chatbot interface to alert them of the potential fraud. Insurance company reviewers can then use the chatbot to review the claim details, perform further investigation, and decide whether to approve or reject the claim. This chatbot-based reviewer workflow is essential to ensure human oversight remains part of the decision loop.

**Algorithm 6:**
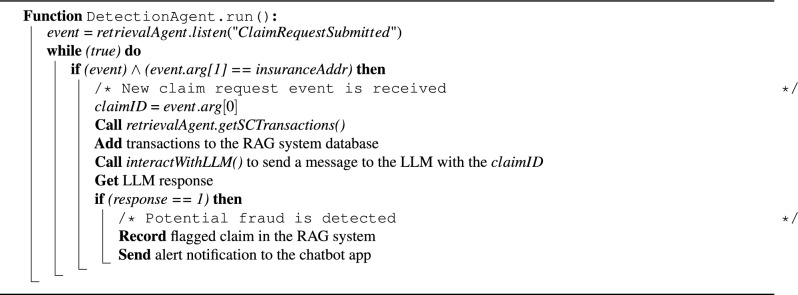
Detecting fraudulent claims in real-time.

## Testing and validation

This section details the testing and validation process conducted to ensure the accurate deployment and execution of functions and algorithms, and verify that outcomes align with expected behavior. We performed a series of tests on the SCs and the LLM to validate their functionality and effectiveness in detecting various types of fraud in health insurance claims.

### Smart contracts testing

We deploy our SCs on the Sepolia testnet for testing purposes, and the Ethereum addresses of the deployed contracts are listed in Table 1. These addresses can be used to verify and review all transactions conducted during the testing phase on the Sepolia testnet explorer^37^.

**Table 1:** Ethereum addresses of the deployed SCs.

SC	Ethereum Address
Registration SC	0xDf9cc3308Eb28CB50279C9b245C94CD837f1b4Ba
MedicalRecords SC	0x34c5B629747d01041665664BD904032e4704fC2b
InsuranceClaims SC	0x2F7BFA23d05db8f1ee8e0F596B738811dC87d736

**Fig. 5:**
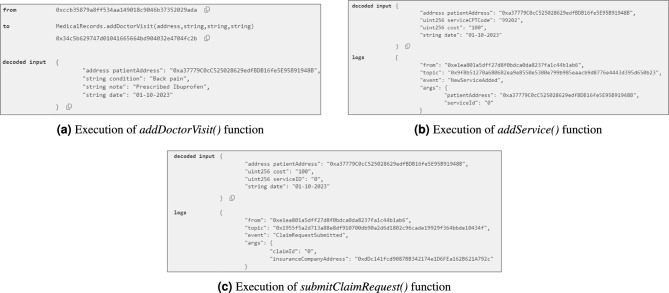
Output logs illustrating the successful execution of SC functions used to record a doctor visit, add medical services, and submit an insurance claim request by the hospital for the rendered services.

**Registering participants and adding patient records:** After deployment, the Registration SC is used to register all participants in the system using different functions based on user role. Then, the MedicalRecords SC is used to add patient medical records. For example, Patient1 visited a clinician for back pain and was prescribed Ibuprofen as treatment. This visit is recorded using the *addDoctorVisit()* function, where the patient’s address, condition, and clinician’s note are added, as shown in Fig. 5a. Similarly, the *addTestResult()*, *addSurgery()*, and *addChronicDisease()* functions are called as needed to add other patient records over time.

**Adding services and prescriptions:** When the visit is done, the clinician records the visit and provided services via the InsuranceClaims SC. The clinician uses the *addService()* function by providing the patient’s address, service CPT code, and service cost. This function then triggers an event, as shown in Fig. 5. The *addPrescription()* function is also called to add the prescription details. Lastly, the pharmacist calls the *dispensePrescription()* to confirm that the patient received the prescribed medication. This function is essential as the pharmacy cannot request a claim unless the prescription has been dispensed to the patient.

**Submitting claim requests:** After services are performed and added to the SC, the pharmacy or hospital where the service was rendered uses the *submitClaimRequest()* function by entering the patient’s address, service ID, and claim amount. This function emits an event with the claim ID, as shown in Fig. 5c. Upon receiving a new claim request, the insurance provider uses the LLM to analyze it for potential fraud. Then, the *approveClaimRequest()* and *rejectClaimRequest()* functions are called as needed based on the claim details.

**Fig. 6:**
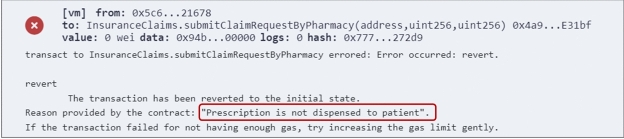
Error message for undispensed prescription.

**Preventing fraudulent claims with SCs:** The SCs are designed to prevent certain types of fraudulent claims without relying on the LLM. This includes duplicate claims, where more than one claim is submitted for the same service. This is prevented by checking the *claimRequested* value for the service, which is set to *true* after submission of the first claim. Additionally, claims for prescriptions not yet dispensed are prevented by checking the *prescriptionDispensed* value of the prescription ID. Finally, the requested claim amount is verified against the total service amount to prevent overcharging. Figure 6 shows an example of an error message displayed when any of these types of fraud is detected.

### LLM testing

Here, we test the LLM capabilities in detecting fraudulent claims in real-time and generating accurate and relevant responses based on extracted blockchain records. We test the LLM’s ability to detect various types of fraud that cannot be detected by SCs, as well as its explainability, reasoning, summarization, and analysis capabilities. To facilitate efficient testing, we have created several testing scenarios targeting different types of fraud and added the corresponding records and details to the SCs.

**Fig. 7:**
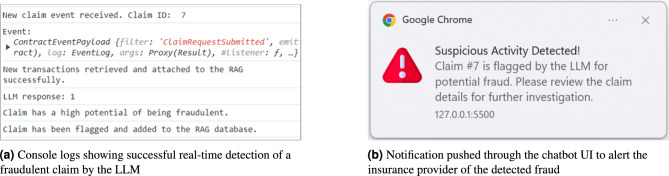
LLM-based real-time detection, flagging, and alerting of a fraudulent claim

**Detecting fraudulent claims in real-time:** Here we test the LLM’s ability to analyze claim requests in real-time and immediately flag any fraudulent ones. Figure 7a illustrates the LLM successfully detecting and flagging a fraudulent claim. Upon receiving the claim event, new transactions are retrieved and added to the vector store. A message is then sent to the LLM, which analyzes the claim and responds with “1”, indicating that a potential fraud has been detected. Subsequently, a notification is pushed to the chatbot UI to alert the insurance provider about the detected fraud, as shown in Fig. 7b.

**Fig. 8:**
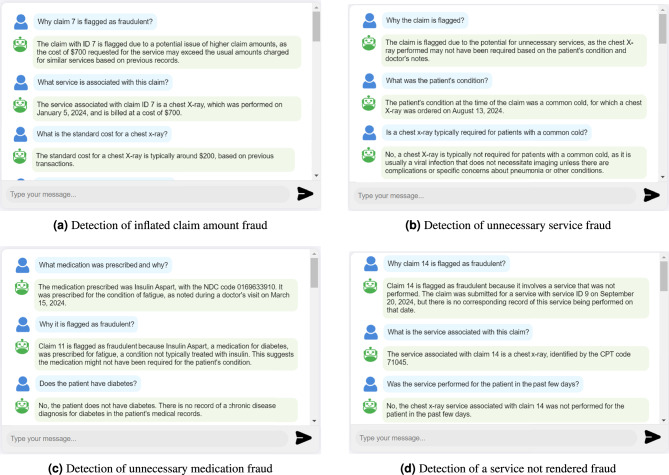
Interaction logs displaying LLM’s successful detection of four types of health insurance fraud.

**Detecting various types of fraudulent claims:** The LLM in our system can detect various types of fraudulent claims, as shown in Fig. 8. This includes duplicate claims, inflated claim amounts, patient-paid services, services not rendered, and unnecessary services and medications. For example, in the case of claim 7, a service that typically costs $200 is submitted with a claim amount of $700, inflating the actual cost to defraud the insurance provider. Figure 8a demonstrates the LLM’s ability to detect this type of fraud successfully. Moreover, Figures 8b and 8c demonstrate the LLM’s ability to detect fraudulent claims involving unnecessary services and medications. In such cases, a clinician orders tests or medications that are not required for the patient’s condition to profit from insurance. This type of fraud is very common and challenging to detect without a thorough review of patient medical records and conditions. Another claim is submitted for an X-ray test that was never conducted on the patient, attempting to receive insurance reimbursement for a service that was not rendered. Figure 8d illustrates the LLM capability to detect this fraud by verifying whether the test record was added to the patient’s records.

The system was tested on six scenarios featuring different types of fraudulent claims. It successfully detected each case and provided clear explanations for all instances of fraud. Full details and chat snapshots of these testing scenarios are available on GitHub at: https://github.com/rubak3/InsuranceFraudDetection.

**Fig. 9:**
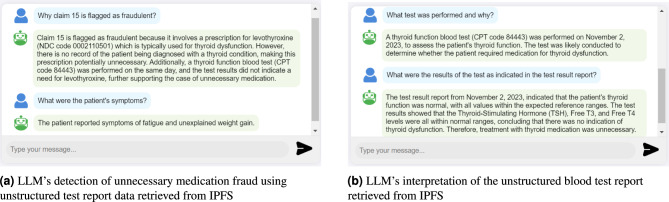
LLM’s analysis of a fraudulent claim using IPFS-stored test report.

**Analyzing Off-Chain Clinical Documents:** In addition to handling structured on-chain data, our system also supports the integration of unstructured clinical documents through IPFS off-chain storage. To test this functionality, we uploaded a sample blood test report to IPFS and stored its hash reference on the blockchain via the MedicalRecords SC. The document was then successfully retrieved and uploaded to the RAG component for analysis. This enables the system to process and interpret clinical notes, lab results, and diagnostic images in natural language, expanding the types of fraud that can be detected, such as misdiagnosis, fabricated reports, and inconsistencies between reported symptoms and treatments. Figures 9a and 9b illustrate the LLM’s ability to interpret and explain an unnecessary medication fraud based on unstructured test result data retrieved from IPFS.

**Fig. 10:**
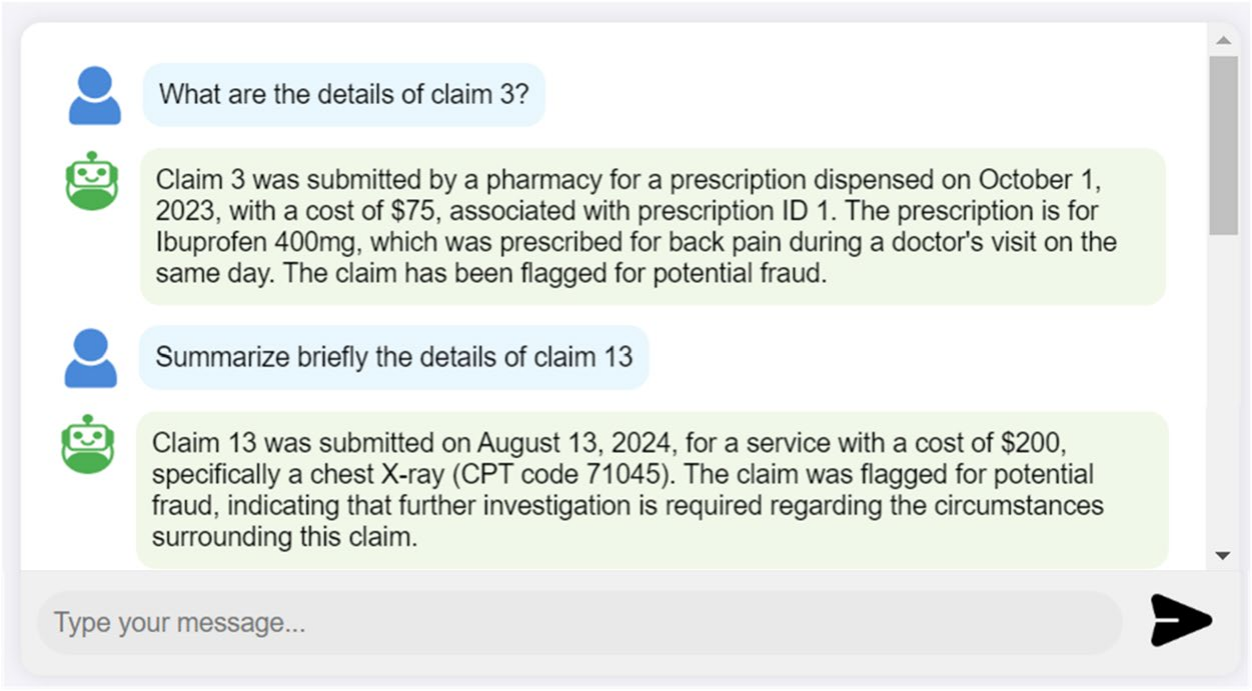
Interaction log displaying LLM-generated summaries of claim details.

**Offering explanations, summaries, and analytical insights:** The LLM exhibits advanced capabilities in reasoning, analysis, explanation, and summarization. These features significantly enhance our system performance in detecting fraud claims, interpreting complex queries, and providing insightful responses. Through its reasoning and analytical abilities, the LLM can assess claims, identify patterns of fraud, and evaluate the context of each case. It also delivers detailed explanations of its findings, so that insurance providers understand the rationale behind its assessments. As demonstrated in figures 8a-8d, the LLM analyzes claims and transaction data from the blockchain and explains the reasons behind the detected fraudulent claims. Additionally, the LLM summarization capabilities, as shown in Fig. 10, effectively condense extensive claim data into concise overviews, which makes it easier for users to grasp critical information quickly.

**Fig. 11:**
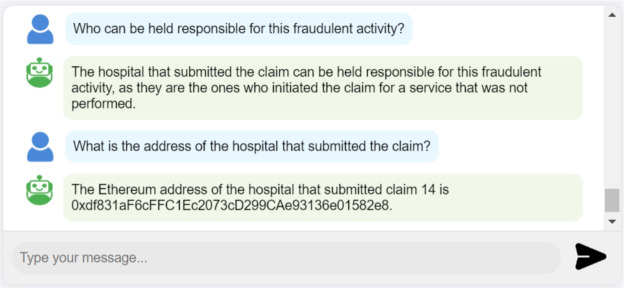
Interaction log showing LLM’s successful identification of the responsible entity behind fraudulent Claim 14.

**Identifying responsible actors in fraudulent claims:** The system leverages the reasoning and analytical capabilities of the LLM, alongside reliable data retrieved from SCs, to effectively identify the responsible entity behind each fraudulent claim, as illustrated in Fig. 11. The accuracy of this data is ensured by the underlying blockchain technology, which guarantees immutability, integrity, and accountability. Each action performed within the system is meticulously logged, creating a reliable audit trail that links users to their transactions.

**Fig. 12:**
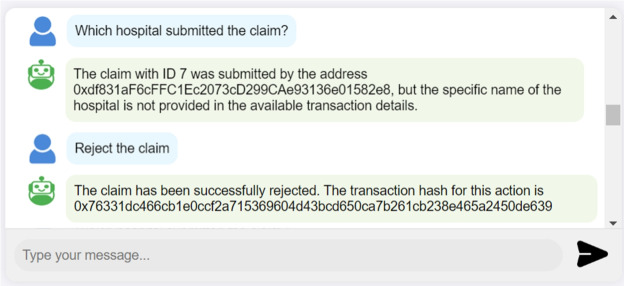
Interaction log showing a SC function called by the LLM to reject a claim based on the user’s query.

**Automating SC function calls:** The AI assistant in our system is equipped with a function calling tool to automatically invoke SC functions based on user requests. As shown in Fig. 12, the user can query the system to perform actions such as rejecting a claim request. In this scenario, the LLM identifies the appropriate SC function, determines the necessary parameters, and executes the function. The system prompts the user to sign this transaction using their connected wallet to ensure security and verify the user’s approval.

## Discussion and evaluation

This section discusses the results and analyzes various aspects of our proposed system. We begin by evaluating how the system meets the main project objectives, followed by an evaluation and discussion of the LLM performance in fraud detection. We further conduct detailed cost, security, and scalability analyses to evaluate the system’s practicality, robustness, and performance under realistic operational conditions. We also highlight the advantages of our solution by comparing it with existing approaches, discuss how it can be generalized for other applications, and address its key challenges and limitations.

### System objectives evaluation

First, we evaluate the performance of our proposed system by examining how it achieves the primary objectives outlined in this paper and addresses the research gaps identified in the literature review. Specifically, we assess how the system ensures data integrity, transparency, and accountability through its blockchain-based framework, as well as how it leverages the integrated blockchain gateway and RAG for efficient blockchain data extraction and retrieval. Additionally, we focus on the system’s ability to process unstructured data, enable real-time detection of various fraud types, and provide intelligent user interaction features.

**Ensuring data integrity, transparency, and accountability:** Our blockchain-based framework ensures the integrity, immutability, traceability, and transparency of medical records and claim details via the SCs. An immutable record of all interactions is created, with each step of the claim process linked to the responsible entity. This also enhances accountability, enabling the identification of individuals responsible for fraudulent claims. Moreover, the SCs facilitate the prevention of certain types of fraud, as actions cannot occur without meeting predefined conditions.

**Automating blockchain data extraction:** The integrated blockchain gateway facilitates the automated extraction of medical records and claim details in real-time. Whenever a new claim request event is received, the system retrieves all associated transactions and attaches them to the RAG database for analysis, eliminating the need for manual data extraction from the blockchain.

**Handling unstructured textual data and enabling real-time fraud detection:** This is achieved through the integration of the LLM and RAG models, which allows the system to effectively analyze unstructured textual data, such as natural language queries and detailed patient records. This eliminates the costly and intensive preprocessing operations typically required for structured data. Moreover, the system continuously monitors claim events, with the LLM analyzing retrieved transactions to flag potential fraud. When fraud is detected, a chatbot notification alerts insurance providers, which saves time and minimizes manual review of all received claims.

**Enabling intelligent user interactions:** The LLM-powered chatbot enables insurance providers to interact with the system using text-based queries. By using blockchain data as the foundation for the LLM responses, the LLM can offer reliable information on claim requests and patient records. It also assists in further investigations by generating clear and relevant summaries and explanations. Additionally, the chatbot automates the process of adding new data to SCs by interpreting conversations and identifying when SC functions need to be called based on user requests.

**Enabling the detection of various types of claim fraud:** The proposed system has demonstrated its effectiveness in detecting various fraudulent activities, including duplicate claims, inflated costs, patient-paid services, services not rendered, and unnecessary procedures or medications. Through the integration of textual blockchain data and the analytical abilities of the LLM, the system thoroughly assesses claims and detects fraudulent activities.

### LLM performance evaluation

The findings presented in Section demonstrate the LLM’s proficiency in detecting claims and managing complex fraud situations. As discussed earlier, the LLM demonstrated its strength in analyzing claims details, identifying potential fraudulent activities, and providing comprehensive reasoning and explanations for its decisions. By leveraging blockchain-stored patient medical records and claim data, the model identifies inconsistencies or patterns indicative of fraud. Moreover, the LLM efficiently identifies responsible entities, such as clinicians or patients involved in suspicious activities, which helps reviewers take appropriate actions following fraud confirmation.

Given the lack of publicly available datasets on fraudulent health insurance claims, particularly those in unstructured or textual formats, we systematically evaluated the effectiveness of LLMs in insurance fraud detection by creating a synthetic dataset consisting of 100 different insurance claims. These samples were carefully created to include both legitimate and fraudulent cases across a wide range of fraud types, such as duplicate billing, inflated costs, services not rendered, and unnecessary procedures or medications. Each claim included realistic clinical details, patient records, and blockchain-style transaction metadata to mimic the data structure handled by our system. The full dataset is publicly available on GitHub (at: https://github.com/rubak3/InsuranceFraudDetection) for reproducibility and further benchmarking. This dataset served as a controlled benchmark to assess the performance of the models in both fraud detection and classification tasks. We evaluated three leading LLMs, GPT-4o, Gemini 1.5 Pro, and Claude 3.5 Sonnet, by prompting them with the claim sets and recording their responses in structured JSON format. As shown in Table 2, all models achieved strong performance in detecting fraudulent claims, with GPT-4o achieving the highest fraud detection accuracy of 99%, followed by Gemini (98%) and Claude (97%). All models maintained perfect precision of 100%, which indicates that no false positives were introduced during evaluation. However, slight differences were observed in recall, with GPT-4o detecting the broadest range of frauds (98.4%), followed by Gemini (96.9%) and Claude (95.3%). In terms of fraud type classification, GPT-4o again performed best with an accuracy of 97%, while Gemini and Claude followed closely with 96% and 95%, respectively. These results suggest that all three models are capable of not only detecting fraud, but also correctly identifying the nature of the fraudulent behavior. GPT-4o demonstrated a stronger ability to reason over contextual data, such as patient diagnoses, medication records, and cost justifications, leading to more accurate classification across edge cases. Overall, these findings support the use of advanced LLMs, particularly GPT-4o, as reliable agents for automating and scaling fraud analysis within blockchain-based insurance systems.

**Table 2:** Fraud detection and classification performance of different LLM models.

Model	Fraud Detection Accuracy	Precision	Recall	F1 Score	Fraud Type Classification Accuracy
GPT-4o	99%	100%	98.4%	99.2%	97%
Gemini 1.5 Pro	98%	100%	96.9%	98.4%	96%
Claude 3.5 Sonnet	97%	100%	95.3%	97.6%	95%

In addition to our custom synthetic benchmark, we further evaluated the accuracy of the proposed LLM-based system using a publicly available clinical dataset focused on detecting fraudulent ICU admissions^38^. This dataset is fully anonymized and publicly released for non-commercial academic research purposes via Kaggle. It does not contain any personally identifiable information (PII) or protected health information (PHI), and all clinical records are de-identified in compliance with data privacy best practices. Therefore, the use of this dataset in our study does not require Institutional Review Board (IRB) approval. From this dataset, we selected 1,425 patient instances covering 30 clinical features for our evaluation, including information on chronic diseases, vital signs, blood test results, and blood gases. This dataset is designed to evaluate whether ICU admission was medically necessary. Although it does not cover the full scope of insurance fraud, it addresses a key fraud type involving unnecessary medical services or medications, which is highly relevant to our use case. To align with our LLM-based setup, we converted the structured records into natural language prompts. The model achieved 95.8% accuracy in identifying potential overtreatment cases, demonstrating the model’s strong ability to analyze and interpret clinical scenarios, particularly in identifying cases of potential overtreatment. This is crucial for detecting fraud types involving unnecessary services or medications, where treatments are provided without sufficient medical justification just to benefit from insurance reimbursement. Compared to the traditional ML approach used in^26^, which reported 88.31% accuracy using a Random Forest classifier on the same dataset, our LLM-based system offers both improved performance and greater interpretability through natural language understanding.

Despite the LLM’s strong performance, it may occasionally generate false positives or false negatives. False positives occur when legitimate claims are incorrectly flagged as fraudulent, while false negatives may arise when fraudulent claims are undetected. These challenges are inherent in any ML model and highlight the importance of human oversight in the fraud detection process. To mitigate these issues, the final decision on fraud detection in our proposed system is left to the insurance company reviewers, who can use the chatbot to conduct further investigations and validate the claims flagged by the LLM. However, some of these errors may also result from hallucinations, where the LLM generates plausible but incorrect interpretations due to the lack of training on fraud-specific cases. We recognize that fine-tuning the LLM on historical fraud patterns could help reduce both false positives and hallucinations by improving the model’s contextual accuracy and domain alignment. This is proposed as an important direction for future enhancement. Overall, even in its current configuration, the LLM significantly enhances the efficiency of fraud detection workflows by allowing reviewers to focus on claims identified as suspicious, thus saving time and resources while maintaining high standards of accuracy and oversight.

The strong performance results also reflect the architectural advantages of using a large LLM over traditional models. While gradient-boosted decision trees (GBDT) and smaller, fine-tuned transformer models may offer competitive performance in structured prediction tasks, they remain limited in scope when applied to the complex and heterogeneous nature of health insurance fraud detection. For example, GBDT models rely on handcrafted features derived from structured datasets and are highly effective when input data is well-labeled and tabular. However, they are incapable of processing free-text clinical notes, physician justifications, or unstructured claim narratives without extensive preprocessing and feature extraction pipelines. Similarly, smaller transformer models, such as DistilBERT and BioBERT, can be fine-tuned on domain-specific datasets to perform classification or named entity recognition with lower computational overhead. However, these models typically require task-specific supervision and perform best in narrowly defined use cases. In contrast, our system employs a large-scale LLM integrated with a RAG framework, which enables it to interpret and reason over long and diverse documents, including blockchain-stored claim histories and unstructured IPFS-stored medical records, without the need for extensive labeled training data. Additionally, the LLM’s capabilities extend beyond pattern recognition as it can generate contextual explanations, summarize patient case histories, and interact with users in natural language, thereby supporting transparency and human-in-the-loop validation. These abilities are not natively supported by smaller models or GBDT classifiers. Moreover, the LLM can generalize across different fraud types based on semantic reasoning, rather than depending solely on statistical correlations learned during training. While smaller models offer advantages in speed, cost, and training efficiency, their limited capacity for contextual reasoning, integration with real-time data streams, and natural language interaction makes them less suitable for end-to-end insurance fraud analysis at scale. Our use of an LLM addresses these limitations and introduces a more adaptive, explainable, and interactive solution, particularly critical in domains like health insurance where both structured and unstructured information drive decision-making.

### Response time and dcalability analysis

To assess the performance of the proposed system in supporting real-time healthcare operations, we conducted a detailed analysis of the execution time for key system functionalities, including SC interactions, transaction retrieval and processing, and interactions with the LLM. The objective is to confirm that the system can consistently deliver timely responses under realistic operational conditions. Table 3 summarizes the average response times and outlines the specific steps involved for each functionality. The results show that the system consistently achieves low response times across all evaluated components, with all functionalities completed in less than 15s. Among these functionalities, real-time fraud detection exhibits the longest response time at approximately 12.94s. This process encompasses detecting a new claim submission event, retrieving recent SC transactions, formatting and uploading these transactions to the LLM’s vector store, and performing a fraud detection query. Despite including multiple stages, the total response time remains minimal and within an acceptable range for automated real-time analysis. Compared to traditional manual claim reviews, this automated workflow significantly accelerates detection and enables the system to flag suspicious claims almost instantly. For LLM interactions, response times vary slightly depending on the nature of the query. When performing a contextual query using previously uploaded transactions (RAG-based), the average time is approximately 6.97s. Queries that involve reading from SCs complete in around 9.48s, while writing to SCs, which includes the overhead of user signature and blockchain propagation, takes 12.32s on average. The capability to interact with SCs through natural language queries and receive rapid responses significantly enhances usability and eliminates the need for manual data inspection or direct SC interaction. Finally, the process of retrieving, processing, and uploading transactions takes approximately 11.11s. This step is typically performed once per batch of new data. This speed demonstrates the system’s ability to retrieve and prepare transaction data quickly, supporting fast and responsive analysis.

**Table 3:** Response time for key system functionalities.

Functionality	Response Time	Process Description
Transactions retrieval	11.11s	Fetching SC transactions + decoding and formatting data + uploading transactions to RAG vector store
LLM query (RAG-based)	6.97s	Sending query to OpenAI API + receiving response based on attached context
LLM query (read from SC)	9.48s	Sending query to OpenAI API + executing SC view function + receiving response
LLM query (write to SC)	12.32s	Sending query to OpenAI API + executing SC write function + signing with user wallet
Real-time fraud detection	12.94s	Receiving claim event + retrieving recent transactions + uploading to RAG + analyzing with LLM + notifying user

Moreover, to assess the scalability of the proposed system under increased operational demands, we conducted stress tests across the main system components. Specifically, we evaluated the system’s behavior under varying levels of concurrent requests, transaction volumes, and knowledge-base sizes. The results presented in Table 4 demonstrate stable and efficient performance across all tested conditions. For SC executions, the values 1-20 refer to the number of concurrent transactions submitted by different users. The observed response times ranged from 11.22s to 13.24s, with all values falling within the expected range for Ethereum network latency, which is typically between 12s and 15s. This confirms the system’s ability to process multiple simultaneous calls efficiently without performance degradation. For transactions retrieval, the numbers indicate the total number of blockchain transactions fetched, processed, and uploaded to the RAG database in a single batch. As the volume increased from 1 to 100 transactions, response time grew slightly from 2.01s to 3.47s. This minimal increase demonstrates that the system handles larger volumes of transaction data efficiently, without introducing significant delay. Lastly, in the case of LLM interactions, the scale corresponds to the number of attached transaction files available to the model at query time. Even as the number of files increased from 1 to 100, the response time remained stable, ranging from 7.88s to 8.82s. This indicates that the LLM can reason over larger knowledge contexts with no performance degradation. This also ensures that even under high loads and with a growing number of blockchain transactions, real-time fraud detection remains fast and effective.

**Table 4:** Average response time for key system functionalities under varying workload conditions.

	1	5	10	20	50	100
Smart contract executions	11.22s	12.46s	11.26s	13.24s	-	-
Transactions retrieval	2.01s	2.47s	1.97s	2.32s	2.72s	3.47s
LLM Interactions (RAG-based)	7.99s	8.29s	8.78s	7.88s	8.82s	8.57s

Overall, these results highlight the system’s ability to maintain low latency and stable performance under varying loads. The consistent behavior across all components confirms that the solution is scalable and well-suited for real-time operations involving high transaction volumes. The current evaluation was conducted under controlled resource constraints; however, in scenarios with significantly higher loads, potential scalability challenges may arise. To mitigate these, Layer 2 blockchain solutions can be utilized to reduce SC execution latency. On the LLM side, limiting the context to recent transactions and periodically discarding older data can help maintain efficient query performance while preserving relevance and accuracy.

### Cost analysis

This subsection analyzes the costs associated with integrating the SCs and the LLM into our system. The objective is to evaluate their impact on overall operational costs by considering execution costs, transaction fees, interaction fees, and resource consumption.

#### Smart contracts cost analysis

Execution costs on the Ethereum network, commonly referred to as gas fees, represent the costs incurred when executing transactions or SC functions. Gas is a unit that measures the computational work required to process and validate transactions on the Ethereum blockchain. The total cost is calculated by multiplying the gas price by the amount of gas used for a particular operation. Factors influencing gas usage include the complexity of the SC, the number of state changes it makes, and the current level of network congestion^39^. The SCs developed in our solution are compatible with any Ethereum Virtual Machine (EVM)-based blockchain, which allows deployment on a wide range of platforms, including Ethereum Mainnet and Layer 2 (L2) solutions. For cost analysis, we use the same gas consumption values across all platforms, as they depend on SC logic rather than the specific network. However, final transaction costs differ due to variations in gas prices and fee models across networks. Table 5 summarizes the estimated costs for executing core SC functions on Ethereum, Arbitrum, and zkSync. These costs are calculated using gas prices and Ether market rates as of July 20, 2025^40^. It is important to note that transaction fees can vary significantly, influenced by fluctuations in Ether market price and the level of congestion on the network.

**Table 5:** Execution costs of the SC functions.

Function Name	Transaction Gas	Cost on Ethereum (USD)	Cost on zkSync (USD)	Cost on Arbitrum (USD)
*registerPatient()*	76927	0.610	0.013	0.003
*addDoctorVisit()/addTestResult()*	124301	0.986	0.021	0.005
*addChronicDisease()/addSurgery()*	77851	0.617	0.013	0.003
*addService()/addPrescription()*	128441	1.019	0.022	0.005
*dispensePrescription()*	79175	0.628	0.013	0.003
*submitClaimRequest()*	143934	1.141	0.024	0.005
*approveClaim()/rejectClaim()*	30362	0.241	0.005	0.001
*payService()/payPrescription()*	46793	0.371	0.008	0.002
*payClaim()*	45302	0.359	0.008	0.002

The analysis reveals variations in gas usage and execution cost across different functions. The *submitClaimRequest()* function has the highest gas consumption and execution cost due to the numerous conditions checked and the need to update multiple variables and mappings. These updates and checks are essential to validate claims and prevent fraud efficiently. Similarly, the functions for adding services and medical records also incur high costs because of their complexity compared to other functions. In contrast, the remaining functions have lower costs, especially the functions for approving and rejecting claim requests, which only update the claim status. However, the fluctuating price of Ether in USD poses a significant challenge for deploying SCs and executing functions, especially during periods of high prices. To address these high costs, deploying SCs on a private blockchain is an effective solution, as it allows for the configuration of gas prices to zero, enabling transactions without incurring any fees. In addition, this approach enhances data privacy and improves transaction efficiency and control over the network. Alternatively, L2 scaling solutions such as zkSync Era and Arbitrum offer significant cost reductions while preserving Ethereum compatibility. Both platforms are fully EVM-compatible and process transactions off-chain before submitting proofs to Ethereum for settlement^41,42^. zkSync leverages zero-knowledge proofs (zk-rollups), while Arbitrum uses optimistic rollups with fraud-proof mechanisms. These architectures allow for substantial savings in transaction fees while maintaining high throughput and strong security guarantees. As illustrated in Table 5, deploying our system on zkSync or Arbitrum can reduce execution costs by over 95% compared to Ethereum Mainnet.

#### LLM cost analysis

Integrating an LLM model into our system incurs operational costs that may impact the system’s scalability. Therefore, it is crucial to evaluate the financial aspects of the LLM integration. The costs associated with implementing an LLM service, such as those from OpenAI, Google, or Anthropic, vary based on the specific model selected and the features used, such as OpenAI’s file search feature used in our testing implementation. These models differ in their pricing structures, capabilities, processing speeds, and performance, which can significantly affect the overall efficiency and effectiveness of our system. Charges are typically calculated based on token usage for the chosen model, where token usage is determined by both input and output tokens, with one token roughly equivalent to 0.75 words. Additionally, we employ a RAG system, which retrieves relevant transaction data from the vector database based on the user query. The retrieved data is converted into tokens and sent to the LLM along with the user query. Consequently, the tokens generated by the RAG retrieval process should be included as part of the input tokens when calculating the total cost.

Table 6 summarizes the average costs associated with integrating different powerful LLM models within our system. The analysis reveals that interaction costs are minimal, with each session costing a maximum of $0.1 even when using the most advanced models. To estimate these costs, we analyzed the maximum number of input and output tokens processed per session during the testing phase using the OpenAI tokenizer tool^43^. In each session, the system processed a maximum of 33K input tokens, including tokens retrieved by the RAG system, and generated up to 400 output tokens. Additionally, we calculated the cost per instance for the real-time analysis of new claim requests, which consistently requires around 9K input tokens and 1 output token, resulting in a cost of less than $0.03 per claim.

**Table 6:** Costs associated with integrating different LLM models.

	GPT-4o^44^	Gemini 1.5 Pro^45^	Claude 3.5 Sonnet^46^
Input Token Cost	$0.0025 / 1K tokens	$0.00125 / 1K tokens	$0.003 / 1K tokens
Output Token Cost	$0.01 / 1K tokens	$0.005 / 1K tokens	$0.015 / 1K tokens
Interaction Session Total Cost	$0.087	$0.043	$0.10
Real-time Detection Total Cost	$0.022	$0.011	$0.027

Regarding the costs associated with the file search tool integrated into our system using OpenAI, the charges depend on the total file size stored. Each transaction in our system is approximately 500 bytes, and the first 1 GB of storage is provided for free by OpenAI. This allows for nearly 2000 transactions before any additional costs are incurred. If the number of transactions exceeds 2000, an additional $0.10 will be charged per day for every additional 2000 transactions. This cost remains relatively low, especially when compared to the substantial annual financial losses associated with fraud. However, to manage costs when transactions size become very high, the system can be adjusted to retain only recent transactions by periodically deleting older ones.

### Smart contracts security analysis

**Fig. 13:**
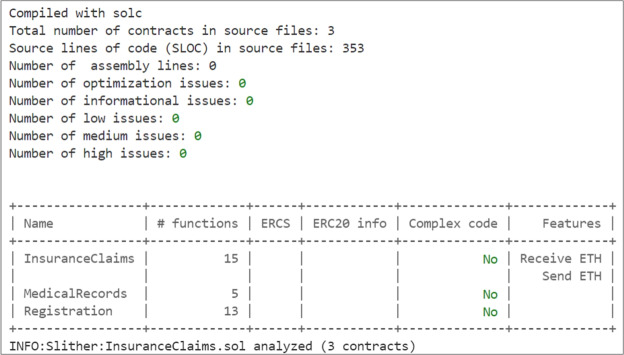
Output of Slither tool showing vulnerability-free SCs.

Blockchain technology is recognized for its security elements, like decentralization and cryptographic hashing methods, along with consensus mechanisms that work together to establish a platform for storing data and conducting transactions securely to uphold the integrity and immutability of information within the system. Nevertheless, despite these capabilities, SC code may still be susceptible to vulnerabilities. As a result, it is crucial to perform security checks to uphold the security of the system and prevent any possible breaches. To evaluate our SCs, we used the Slither analysis tool, a static analysis framework designed for Solidity contracts. Slither effectively detects vulnerabilities by offering comprehensive visual insights into the contract structure and flagging potential weaknesses along with their levels of severity^47^. Our assessment revealed a few low-risk issues related to best practices, particularly in variable naming conventions, which were resolved by updating the code to adhere to established naming standards. A subsequent analysis shown in Fig. 13 confirmed that our SCs are free from vulnerabilities at all severity levels. This result confirms the reliability of our SCs and assures that they operate securely without introducing any security issues.

### Comparison with existing solutions

Tables 7 and 8 compare our proposed solution with the existing solutions in the literature. These comparisons highlight the key advantages of our solution for health insurance fraud detection, showcasing its novelty in integrating blockchain technology with LLM-based real-time detection and intelligent user interactions.

Blockchain-based solutions primarily focus on secure storage, traceability, and process integrity using SCs and immutable ledgers. While these frameworks succeed in ensuring tamper-proof claim handling, they typically rely on manual processes or rigid rule-based fraud checks embedded in SCs. As shown in Table 7, none of these systems support unstructured clinical data, such as physician notes or scanned documents, which significantly limits their fraud detection capabilities. Moreover, they lack intelligent analytics and interactive features, resulting in detection processes that are static, slow, and not adaptable to diverse or evolving fraud scenarios. They also do not support real-time detection or automated retrieval of data from the blockchain for integration with external analytical engines. For example, studies such as^13–15^ depend on manual identification or SC-based validation logic, which cannot reason over nuanced fraud patterns or accommodate varied data types.

In contrast, the ML-based and hybrid solutions presented in Table 8 demonstrate improved detection performance using a range of supervised and unsupervised models. These approaches can classify or flag anomalous claims more effectively than manual or SC-based review. For instance,^21–24^ employ models such as Random Forest, XGBoost, and K-Means to detect common fraud types like duplicate claims, upcoding, or suspicious billing. However, these models are typically trained on structured data only and lack the ability to process unstructured inputs. Additionally, they operate independently of blockchain infrastructure, and therefore do not benefit from data immutability, auditability, or decentralized access control. Most solutions also lack real-time analysis, intelligent user interaction, or explainability features, and they do not integrate RAG or LLMs to enhance reasoning over claim details.

Our proposed solution addresses the limitations of prior blockchain and ML-based systems through a unified, intelligent framework. It uses Ethereum SCs for tamper-proof claim storage and integrates IPFS to support both structured and unstructured clinical data, overcoming the data-type restrictions of earlier approaches. To enable real-time, context-aware fraud detection, we incorporate a RAG pipeline powered by an LLM, which automatically retrieves relevant on-chain and off-chain data and reasons over complex fraud scenarios such as unrendered services and unnecessary treatments. This enables the detection of a broader range of fraud types, unlike prior approaches that are restricted to predefined cases. Furthermore, the LLM-powered chatbot enables natural language interaction for querying, explanation, and summarization of claim details. This enhances transparency, speeds up fraud resolution, and reduces false positives. Together, these features deliver a comprehensive and adaptive fraud detection solution that directly addresses the key shortcomings of existing methods.

### Generalization

Our proposed system, with its core technologies and features, is capable of addressing fraud detection challenges across various sectors beyond health insurance. It offers innovative and superior capabilities, including real-time detection, LLM-powered user-friendly interactions, and highly adaptable analytical and reasoning capabilities. The system’s modular architecture, depicted in Fig. 2, supports seamless integration with different LLMs, whether open-source, proprietary, or custom-developed. This flexibility also enables organizations in other sectors to adopt the system with minimal amendments and adaptations to the SCs, ensuring they accurately reflect the specific requirements and regulatory obligations of each use case.

The financial sector is one example of where this system could be applied. It can be adapted to improve fraud detection in areas like loan applications, identity verification, and credit card transactions. By combining real-time monitoring with LLM-powered analysis, financial institutions can detect patterns of fraudulent behavior and flag them for further investigation. In logistics and transportation, the system can monitor shipment records, verify delivery milestones, and detect issues such as fraudulent claims for undelivered goods. By combining SCs with LLM-powered analysis, logistics providers can ensure their delivery data is accurate and resolve disputes quickly and efficiently.

Overall, the system’s modular architecture and implementation algorithms allow for easy and flexible customization to meet regulatory requirements and industry-specific processes. This enables organizations to implement tailored fraud detection and claims validation systems with minimal adjustments.

**Table 7:** Comparison of our proposed solution with existing blockchain-based fraud detection solutions.

Study	SC Used	Unstructured Data Support	Main Features	Detection Automation	Detection Process Detail	Fraud Types Covered
^13^	Yes	No	Policy compliance validation	Manual	Policy compliance checked via predefined SC rules	Policy non-compliance
^14^	Yes	No	Validator-assisted claim review	Manual	Claims reviewed by validators assisted by SC rules	Overbilling, fake services, claim exaggeration
^15^	Yes	No	Blockchain ledger for flagging anomalies	Manual	Manual identification of anomalies, SC logs used for traceability	Service duplication, fake claims
^16^	Yes	No	Layered architecture, SCs for claims and identity	Manual	Conceptual fraud taxonomy, no implemented detection mechanism	Billing, ID theft
^17^	Yes	No	Decentralized ledger, on-chain storage	Automated	SCs validate structured claim formats and track data on-chain	Claim duplication, false billing
^19^	Yes	No	Tamper-proof insurance workflows	Automated	Uses workflow logic for insurance task automation, no content-level fraud inference	Misuse of policy, unauthorized claims
^20^	Yes	No	Multistage claim validation process	Automated	Multi-stage contract logic filters suspicious claims	False billing, impersonation
**Our Solution**	**Yes**	**Yes**	**Blockchain-based real-time detection with LLM reasoning over SC on-chain and IPFS off-chain data**	**Automated**	**LLM retrieves and reasons over on-chain and off-chain data (structured and unstructured), supports interactive, contextual detection and explanation**	**Duplicate claims, inflated costs, patient-paid services, services not rendered, and unnecessary procedures/medications**

**Table 8:** Comparison of our proposed solution with existing ML-based fraud detection solutions.

Study	Blockchain-based	Unstructured Data Support	Real-time Detection & Automated Retrieval	Intelligent User Interactions	Detection Process	Fraud Types Covered	Evaluation Dataset	Accuracy
^21^	No	NA	NA	NA	Data mining + unsupervised models (IF, CBLOF, ECOD, OCSVM)	Abnormal provider behavior, service misuse	NA	NA
^2^	No	NA	NA	NA	RF, LR, ANN trained on labeled claims	Policy misuse, rebilling, upcoding, unbundling	Saudi CHI Claims (Private)	98.2%
^22^	No	NA	NA	NA	XGBoost + rule-based triggers	Duplicate claims, policy misuse, billing	Ayushman Bharat (India) Claims	92.2%
^23^	No	NA	Partial	NA	XGBoost with Bayesian optimization	Generic insurance fraud types	Kaggle Healthcare Provider Fraud (Public)	98%
^24^	No	NA	NA	NA	Supervised ML classifiers on structured data	Billing fraud, eligibility abuse	NA	NA
^26^	Yes	NA	Partial	NA	RF, KNN, SVM + SCs with multisource signatures	Staged illness, inflated billing, fictitious records	Kaggle Covid-19 Clinical Data (Public)	88%
^27^	No	NA	NA	NA	K-means clustering to flag outliers	Upcoding, phantom billing, identity fraud	NA	88%
^27^	Yes	NA	NA	NA	Decision tree rules in SCs	General NHIS fraud types	NHIS Ghana Audited Claims (Private)	97.96%
**Our solution**	**Yes**	**Yes**	**Yes**	**LLM-powered chatbot**	**RAG + LLM on structured and unstructured blockchain data**	**Duplicate claims, inflated costs, patient-paid services, services not rendered, and unnecessary procedures/medications**	**Synthetic Insurance Fraud Dataset**	**99%**

### Challenges and limitations

Despite the advantages of our system in identifying insurance claim fraud, there are still limitations and challenges to overcome. Protecting data privacy presents a key concern, particularly when managing medical records that should only be viewed by authorized personnel. One potential solution involves implementing a private or consortium blockchain, which can restrict data access to verified participants and support compliance with healthcare privacy regulations. In particular, compliance with standards such as the General Data Protection Regulation (GDPR) and the Health Insurance Portability and Accountability Act (HIPAA) requires strict control over data exposure, access logging, and user consent. Since public blockchains may expose metadata and are not suitable for storing PHI, shifting toward private infrastructure is essential for real-world deployments. Furthermore, zero-knowledge proofs (ZKPs) can be explored to verify specific claim conditions, such as the existence of a diagnosis or treatment, without disclosing underlying sensitive data. To further strengthen privacy, future implementations may leverage locally deployed LLMs to ensure that all clinical and claim data remain within the organization’s infrastructure and are not transmitted to external APIs. These combined strategies aim to preserve patient confidentiality while enabling trustworthy, auditable, and regulation-compliant fraud detection. Another limitation lies in the evaluation process. Due to the lack of publicly available health insurance fraud datasets, especially those involving realistic, unstructured claim narratives, our system was primarily assessed using synthetic data. While this allowed us to demonstrate model effectiveness across a range of fraud types, it limits the generalizability of the findings and introduces difficulties in benchmarking against prior work. The lack of standard datasets and metrics makes direct comparison with traditional ML approaches challenging. Future work should prioritize the development or collaborative curation of shared benchmark datasets that reflect real-world fraud complexity and diversity, which would facilitate more robust, transparent, and comparable evaluations across systems. Finally, like all LLM-based systems, ours faces the challenge of potential hallucinations, where the model may generate incorrect analyses or conclusions, resulting in false-positive or false-negative detections. While the LLM shows strong reasoning abilities, its zero-shot configuration limits exposure to fraud-specific terminology and patterns. To address this, future work will explore fine-tuning the model on labeled fraud patterns from various insurance claim types. This would enable the model to recognize a wider range of fraud and reduce potential hallucinations, thus expanding its effectiveness across different scenarios. In addition, we propose a complementary strategy where successful fraud analyses generated by the LLM are stored on IPFS in a structured format. These validated scenarios can be incrementally indexed into the RAG vector store to form a continuously expanding knowledge base to guide future detections. This approach supports self-improving fraud intelligence and enhances the model’s contextual understanding over time.

## Conclusion

In this paper, we have proposed a novel solution aimed at enhancing fraud detection in health insurance claims by addressing the limitations of traditional manual approaches. We integrated blockchain technology and IPFS decentralized storage to securely store structured and unstructured claim details on a decentralized, immutable ledger, ensuring data integrity, transparency, and accountability. This reliable data is then utilized by an LLM agent enhanced with RAG for real-time analysis and detection of various types of fraud, including duplicate claims, inflated amounts, unnecessary services, and services not rendered. The inclusion of a chatbot further enhanced the system’s usability, enabling access to LLM-based features for claim inquiries, summaries, and explanations. The system effectively addressed key research gaps identified in the literature, such as the ability to perform real-time fraud detection and provide intelligent user interactions. The testing scenarios confirmed the LLM’s efficiency in analyzing blockchain transactions, generating context-aware responses, and facilitating automated interactions with SCs. Moreover, evaluation using both synthetic and public datasets showed strong performance, with the LLM achieving up to 99% fraud detection accuracy and perfect precision. Cost analysis revealed minimal integration costs for the LLM services, though higher costs for certain SC functions were noted, which could be mitigated through private blockchains or Layer 2 solutions. Additionally, security analysis using the Slither tool verified the robustness of the SCs against known vulnerabilities, ensuring a secure operating environment. Response time and scalability analysis further demonstrated that our system maintains low latency and consistent performance even under increasing workloads, with the full retrieval, analysis, and fraud detection process completed in just 13 seconds. Our comparative analysis highlighted the novelty of this solution over existing approaches, particularly its adaptability and generalizability to a wide range of industries beyond health insurance, such as financial services, logistics, and manufacturing. For future work, we aim to deploy the solution on a private blockchain to improve privacy and reduce operational costs. Additionally, we plan to fine-tune the LLM to better recognize specific fraud patterns and enable the reporting of historical fraud detection data to further enhance the model’s efficiency and accuracy.

## Data Availability

The source code is publicly available on GitHub at: https://github.com/rubak3/InsuranceFraudDetection.
